# Supplementary material for: Defining the Effect of Oxytocin Use in Farrowing Sows on Stillbirth Rate: A Systematic Review with a Meta-Analysis
Source: Animals (Basel). 2022 Jul 13;12(14):1795. doi: 10.3390/ani12141795 (PMC9311507; doi:10.3390/ani12141795)
Supplement: Supplementary file 1 [file animals-12-01795-s001.zip › animals-1768431-supplementary.pdf]

Table S1. Databases used to search for relevant literature

Database	Interface	Query Box	Wildcards
PubMed	National Center for Biotechnology Information (NCBI)	All fields	* - any group of characters<br>Note: wildcard can only be used if word is 4 or more characters
CAB Direct	CAB Interface (CABI)	All fields	? - 0 to 1 character<br>* - any group of characters
Web of Science Core Collection	Web of Knowledge	Topic (title, abstract, author keywords and Keyword Plus Ò)	$ - 0 to 1 character<br>* - any group of characters
ProQuest dissertations and theses global (1988 - present)	ProQuest	Anywhere except full text	? - 0 to 1 character<br>* - any group of characters

Table S2. Results of initial search string to identify articles investigating oxytocin use at farrowing and stillbirth rates, published between 1970 - present using Web of Science core collection database as of July 23/2020

#	Search Terms	Results
1	(swine OR sow$ OR porcine OR pig$ OR gilt$)	440,312
2	(oxytocin OR carbetocin)	27,205
3	(stillbirth OR dystocia OR farrow* OR “fetal expulsion” OR intrapartum OR parturition OR stillborn OR birth OR meconium OR mortality OR hypoxia)	1,467,554
4	1 AND 2	973
5	3 AND 4	282

Table S3. Controlled vocabulary terms for PICO keywords

Key Term	MeSH 2020 browser:	CAB thesaurus
**Population :**		
Pigs	Sus scrofa	Sus domesticus<br>Sus scrofa domesticus
**Intervention:**		
Oxytocin*	N/A	N/A
Carbetocin*	N/A	N/A
**Outcome:**		
Stillbirth	Fetal death	Fetal death
Dystocia	Uterine inertia	Parturition complications
Hypoxia	Anoxia<br>Anoxemia<br>Hypoxemia	Anoxia
Mortality	Fetal Mortality	Neonatal mortality<br>Perinatal mortality

* Oxytocin and carbetocin names are specified for animal use - no synonyms necessary
**Web of science has no controlled vocabulary or assignment of subject terms
*** Proquest uses MeSH 2020 thesaurus

Table S4. Results of search string with addition of controlled vocabulary, published between 1970 - present using Web of Science core collection database as of July 23/2020

#	Search Terms	Results
1	(swine OR sow$ OR porcine OR pig$ OR gilt$ OR "sus scrofa" OR "sus domesticus" OR "sus scrofa domesticus")	442, 036
2	(oxytocin OR carbetocin)	27, 205
3	(stillbirth OR dystocia OR farrow* OR "fetal expulsion" OR intrapartum OR parturition OR stillborn OR birth OR meconium OR mortality OR hypoxia OR "fetal death" OR "uterine inertia" OR "parturition complications" OR anoxia OR anoxemia OR hypoxemia OR "fetal mortality" OR "neonatal mortality" OR "perinatal mortality")	1,491,102
4	1 AND 2	973
5	3 AND 4	284

Table S5. Time and location characteristics of the 46 eligible studies.

Study	Year of publication	Year (or range) study was conducted	Month(s) Study was conducted	Country study was conducted
[70]	2006	Doesn't specify	Doesn't specify	Doesn't specify
[71]	2010	Doesn't specify	Doesn't specify	Italy
[72]	2000	Doesn't specify	Doesn't specify	Doesn't specify
[50]	2014	Doesn't specify	Doesn't specify	Doesn't specify
[73]	Doesn't Specify	Doesn't specify	Doesn't specify	Doesn't specify
[32]	2005	2005	Doesn't specify	Canada and United States
[74]	2001	1999-2001	January, February, November, December	Greece
[29]	1998	1995-1996	January, February, March, April, May, June, December	Greece
[75]	2016	Doesn't specify	Doesn't specify	Thailand
[76]	2018	Doesn't specify	Doesn't specify	Doesn't specify
[31]	2003	Doesn't specify	Doesn't specify	Canada
[77]	1986	1984	June, July, August, September, October, November	Doesn't specify
[78]	2014	Doesn't specify	Doesn't specify	Doesn't specify
[33]	1987	Doesn't specify	Doesn't specify	Doesn't specify
[35]	1986	Doesn't specify	Doesn't specify	Doesn't specify
[79]	2009	Doesn't specify	Doesn't specify	Mexico
[80]	2009	Doesn't specify	Doesn't specify	Mexico
[36]	1983	Doesn't specify	Doesn't specify	Doesn't specify
[81]	1990	Doesn't specify	Doesn't specify	Doesn't specify
[37]	2019	Doesn't specify	Doesn't specify	Thailand
[38]	2006	Doesn't specify	Doesn't specify	Doesn't specify
[82]	1998	Doesn't specify	Doesn't specify	Doesn't specify
[83]	1995	Doesn't specify	Doesn't specify	Canada
[84]	1990	Doesn't specify	Doesn't specify	Doesn't specify
[40]	2005	2003-2004	January, February, November, December	Mexico
[39]	2002	1998	June, July, August, September, October	Mexico
[41]	2005	Doesn't specify	Doesn't specify	Doesn't specify
[42]	2005	2003	March, April, June, July	Mexico
[43]	2006	2003	March, April, May	Mexico
[45]	2007	2003-2004	January, February, November, December	Doesn't specify
[85]	2014	Doesn't specify	Doesn't specify	Doesn't specify
[47]	1983	Doesn't specify	Doesn't specify	Doesn't specify
[48]	1988	Doesn't specify	Doesn't specify	Doesn't specify

[49]	1987	1984-1986	January, February, March, April, May, June, July, August, September, October, November, December	Australia
[86]	1994	1993-1994	January, February, October, November, December	Doesn't specify
[51]	2019	Doesn't specify	Doesn't specify	Australia
[87]	2005	Doesn't specify	Doesn't specify	Germany
[52]	1984	Doesn't specify	Doesn't specify	Doesn't specify
[53]	1996	Doesn't specify	Doesn't specify	Doesn't specify
[30]	2004	2001	February, March, April, May, June, July	Mexico
[46]	2007	Doesn't specify	Doesn't specify	Doesn't specify
[88]	2009	Doesn't specify	Doesn't specify	Doesn't specify
[89]	2001	Doesn't specify	Doesn't specify	Doesn't specify
[44]	2006	Doesn't specify	Doesn't specify	Doesn't specify
[34]	2006	Doesn't specify	Doesn't specify	Doesn't specify
[90]	1992	Doesn't specify	Doesn't specify	Doesn't specify

Table S6. Experimental design and animal characteristics of the 46 eligible studies.

Study	How many study groups?	Type of Study Allocation	Number of replicates in the study per group	Setting	NSites	Breed of sows	Sample size
[70]	2	Blocked randomization by parity, weight, body condition and farrowing induction	7	Research farm	1	Large White x Landrace	113
[71]	3	Simple randomized	0	Commercial farm	1	Doesn't specify	240
[72]	5	Stratified randomization	0	Commercial farm	1	Doesn't specify	214
[50]	3	Simple randomized	0	Commercial farm	2	Doesn't specify	150
[73]	3	Simple randomized	0	Doesn't Specify	1	Doesn't specify	180
[32]	6	Not described	0	Research farm/Commercial farm	2	Doesn't specify	250
[74]	2	Not described	4	Commercial farm	1	Large White X Landrace	35
[29]	3	Stratified randomizationsows were ran- domly divided into the following three treatment groups, sows of each group were further divided into three subgroups (20 sows per each subgroup}accord- ing to the time of treatment (day 111, 112 and 113)	0	Doesn't Specify	1	Large white X Landrace	180

[75]	3	Simple randomizedNot described	0	Commercial farm	1	Landrace x Yorkshire	183
[76]	3	Simple Randomized	0	Doesn't Specify		Landrace × Yorkshire	183
[31]	4	Not described	0	Commercial farm	1	Doesn't specify	79
[77]	6	Simple randomized	0	Doesn't Specify	1	Yorkshire x Landrace	60
[78]	4	Stratified randomization	3	Commercial farm	1	Topigs 20	118
[33]	11	Stratified randomization2 studies: Study 1: (7 groups) 10 mg of PGF on day 112, 113, 114 or not treated. 20 hours later given 0, 5, 10, 20 or 30 USP U of oxytocin. Study 2: (4 groups) 10 mg of PGF followed by 20 USP of oxyticin at 16, 20 24 hours	0	Doesn't Specify	1	Doesn't specify	292
[35]	5	Not described	0	Doesn't Specify	1	Doesn't specify	94
[79]	4	Not described	0	Commercial farm	1	Yorkshire xLandrace	60
[80]	4	Stratified randomization	0	Commercial farm	1	Yorkshire x Landrace	60
[36]	6	Simple randomized	0	Doesn't Specify	2	German landrace OR German landrace x German large white	165
[81]	6	Simple randomized	0	Doesn't Specify	4	German Landrace, duroc, "tetra"	279
[37]	3	Simple randomized	0	Commercial farm	1	Yorkshire x Landrace	186
[38]	5	Not described	0	Commercial farm	1	Doesn't specify	50
[82]	3	Not described	0	Commercial farm	1	Yorkshire and Landrace	267
[83]	4	Not described	0	Research farm	1	Doesn't specify	43
[84]	5	Not described	0	Doesn't Specify	1	Landrace x Large White	11
[40]	2	Stratified randomization	0	Doesn't Specify	1	Yorkshire × Landrace	120
[39]	3	Stratified randomization randomly assigned to 3 stratified groups of 60 sows each. T	0	Doesn't Specify	1	Doesn't specify	180

[41]	4	Blocked randomization based on pregnancy number (Parity), back fat >26mm excluded	0	Commercial farm	1	Yorkshire x Landrace	200
[42]	3	Blocked randomization by parity (30 per parity from first to sixth parity)	3	Doesn't Specify	1	Yorkshire x Landrace hybrid	180
[43]	6	Blocked randomization Parity 60 for each parity group from one to five (10 of each parity group for each group)	0	Commercial farm	1	Yorkshire × Landrace	300
[45]	4	Simple randomized	0	Commercial farm	1	Yorkshirex landrace	200
[85]	3	Stratified randomization - divided into day 113 or 114 of gestation for the treatment of prostaglandin, more over analysis within group presented for primiparous and multiparous sows	0	Doesn't Specify	1	Camborough hybrid	1258
[47]	6	Not described	0	Commercial farm	6	Doesn't specify	1152
[48]	6	Simple randomized	0	Commercial farm	1	Doesn't specify	119
[49]	4	Stratified randomization	7	Research farm	1	Large White	142
[86]	3	Stratified randomization	0	Commercial farm	1	Landrace x Large white	24
[51]	3	Systematic by parity	4	Research farm	1	Large White x Landrace	102
[87]	3	Blocked randomization by parity	0	Commercial farm	1	German Yorkshire x German Landrace	17
[52]	6	Simple randomized	0	Commercial farm	1	German Landrace and German Yorkshire x Landrace cross-bred	200
[53]	4	Simple randomized	0	Doesn't Specify	1	Landrace-Yorkshire	80
[30]	3	Simple randomized	0	Commercial farm	1	Yorkshire × Landrace	120
[46]	4	Simple randomized	1	Commercial farm	1	Yorkshire-Landrace	200
[88]	4	Simple randomized	0	Research farm	1	Doesn't specify	40

[89]	3	Blocked randomization by dose of oxytocin and primiparous vs pluripartous	0	Research farm	1	Doesn't specify	1339
[44]	2	Simple randomized	0	Doesn't Specify	1	Doesn't specify	80
[34]	5	Not described	0	Doesn't Specify	1	Doesn't specify	1975
[90]	4	Not described	0	Commercial farm	1	Landrace x Large White	116

Table S7. Objectives and categorization of the 46 eligible studies.

Study	Objective category	What was the objective(s) of the study?
[70]	Farrowing assistance	Evaluate the impact of carbetocin at the onset of parturition, on the birth process and piglet survival
[71]	Induction program	To compare 2 synchronization protocols, using Carbetocin and a synthetic oxytocin with Prostiglandin
[72]	Induction program	Examines the efficacy and impact on sow behaviour of cloprostenol and dinoprost to induce and synchronize farrowing.
[50]	Farrowing assistance	Evaluate the use of oxytocin and carbetocin in farrowing sows and its effect on the course of parturition, and the vitality of the neonatal pigs
[73]	Farrowing assistance	Evaluate the effect of two oxytocin products administered at the onset of fetal expulsion on the integrity of piglet umbilical cords, presence of meconium of their skin and the neonatal mortality
[32]	Induction program	Examine the effect of dexamethasone on the farrowing response of the sow and growth of the litter, as well as to determine the incidence of oxytocin associated farrowing problems after induction with a single or split dose of PGF.
[74]	Induction program	Examine the associate Fusarium mycotoxicosis with failure of induction of parturition with PGF2alpha and oxytocin in sows.
[29]	Induction program	Examine the effects of cloprostenol injected on the 111 th. 112th or 113th day of pregnancy, alone or in combination with an oxytocin injection 24 h later, on the induction of parturition during the 8-h working day
[75]	Induction program	Determine gestation length, farrowing time and neonatal piglets characteristic after induction of parturition by using PGF2α or PGF2α in combination with carbetocin in sows.
[76]	Induction program	Examine the efficacy of the combination of PGF2α and carbetocin to induce parturition compared to the use of PGF2α alone and the impact of carbetocin administration on sow reproductive performance
[31]	Induction program	Evaluate the farrowing response to induction with PGF2α either as a single injection or as two injections administered 6 hours apart plus 0 or 20 IU of oxytocin at 24 hours after initial PGF2α injection
[77]	Induction program	Determine if an advantage could be obtained from an injection of 10, 20 or 30 iu of oxytocin after PGF was given on day 112 of pregnancy
[78]	Induction program	Investigate the effects of a split-dose technique with each time half the dose of PG (2×1/2PG) as a farrowing induction protocol in sows on day 114 of gestation, and to compare the effects with the combined use of PG and OT, the single use of PG, and a negative control group.

[33]	Induction program	Determine whether the dose of oxytocin or the interval between PG and oxytocin influences the synchrony of farrowing and the prevalence of intrapartum complications
[35]	Induction program	Determine if the administration of a sequential treatment of estradiol benzoate (EB) and oxytocin, at an appropriate time after PGF2a to permit a decline in progesterone, would improve the predictability and synchronization of induced parturition.
[79]	Farrowing assistance	Evaluate the obstetric and neonatal outcomes to oxytocin 1 IU/12 kg BW administered at advanced stages of parturition to sows with eutocic and dystocic farrowing, with a view to clarifying the clinical criteria when considering oxytocin administration in farrowing sows
[80]	Farrowing assistance	Determine by blood gasometry, the physio-metabolic profile of the sow in eutocic and dystocic farowing, with and without oxytocin administration
[36]	Induction program	investigation was to determine the time of parturition more precisely by injecting oxytocin at a fixed time after PG treatment.
[81]	Induction program	Develop a practical routine procedure to control the time of parturition more precisely
[37]	Farrowing assistance	Evaluate the effects of carbetocin administration, compared to oxytocin, on farrowing duration, birth interval, colostrum production, and piglet survival and vitality
[38]	Induction program	Examine the efficacy of different doses and routes of R-cloprostenol administration on the parturition response in sows.
[82]	Induction program	Determine the effect of a second injection of either cloprostenol or oxytocin after initial cloprostenol induction on the farrowing response of sows.
[83]	Induction program	Determine the efficacy of propranolol for improving the predictability of PGF-induced parturition in sows.
[84]	Induction program	Understand induced deliveries in order to design better suited induction methods.
[40]	Farrowing assistance	Evaluate the effect of oxytocin in sows during parturition on myometrial activity and intra-uterine and post-natal asphyxia.
[39]	Farrowing assistance	Evaluate the effect of 2 oxytocin products administered to sows at the onset of fetal expulsion on the integrity of umbilical cords, meconium staining, and piglet mortality
[41]	Farrowing assistance	Investigate dose minimization of oxytocin administered early during parturition in sows in order to find lower dosages having beneficial uterine effects without fetal complications
[42]	Farrowing assistance	Investigate the clinical effects of vetrabutin chlorhydrate and oxytocin on stillbirth rate and asphyxia variables in swine.
[43]	Farrowing assistance	Evaluate the use of different routes of oxytocin in farrowing sows, and its effect regarding intrauterine and postnatal asphyxia through the identification of fetal hypoxia signs by electronic fetal and uterine monitoring

[45]	Farrowing assistance	Compare various dosages of oxytocin in peri-parturition pigs
[85]	Induction program	Evaluate induction of parturition and duration with PGF either on day 113 or 114 followed 24 hours after by same dose of oxytocin in primiparous and multiparous sows
[47]	Farrowing assistance	Evaluates the effects of polstygmin, oxytocin and prostaglandin on parturition and stillbirths
[48]	Induction program	Measure the efficacy of fenprostalene with and without oxytocin
[49]	Induction program	Evaluate the efficacy of a combined drug treatment regime (PGF-2 alpha and parasympathomimetic) in increasing the total number of piglets that survive to Day 3 post-partum
[86]	Induction program	To determine the efficacy and and consistency of cloprostenol in the induction of farrowing using vulvomucosal route, examine whether the predictability of such induced farrowings is dose dependentand investigate the effects of oxytocin farrowing induction of parturition with cloprostenol by the vulvomucosal route
[51]	Farrowing assistance	Examine whether treatment with carbetocin, compared to oxytocin, would accelerate farrowing without resulting in the adverse effects of oxytocin on piglet viability and survival.
[87]	Induction program	Determine whether the neonatal pH value is influenced by partus induction of sows and whether an influence is exerted on postnatal development of the pH value in piglets.
[52]	Induction program	Determine the lowest effective dose of oxytocin which provided synchronized, timed parturitions without the need for assistance.
[53]	Induction program	Investigate AGN 190851 to determine its effectiveness for indllcing farrowing when combined with PGF2
[30]	Farrowing assistance	Evaluate in penned sows the effect of two commercial oxytocin products administered at the onset of the fetal expulsion period on umbilical cord pathology, degree of asphyxia and intra-partum piglet mortality
[46]	Farrowing assistance	Evaluate whether time of administration of oxytocin during parturition may alter the uterine response and fetal outcomes.
[88]	Induction program	Determine the minimum effective dose of oxytocin as an aid to more specifically induce a synchronized farrowing when using PG with the aim of inducing a programmed farrowing with in working day hours
[89]	Induction program	Examine the use of Deptocin after closprostenol
[44]	Farrowing assistance	Evaluate the effect of teat massage and oxytocin treatment on the delivery outcome and uterine dynamics on maternal-foetal dystocic sows.
[34]	Induction program	Shorten the duration of gestation down to approx. 114 days and to shorten the duration of the process of birth.
[90]	Farrowing assistance	Examines the effects of difference doses of carbetocin on parturition

Table S8. Risk of bias judgement for each domain of the 46 eligible studies.

Study	Domain 1	Domain 2	Domain 3	Domain 4	Domain 5	Overall Risk-of-bias judgement
[70]	Some concern	Low	Some concern	Some concern	Low	Some concern
[71]	Some concern	Low	Low	Some concern	Some concern	Some concern
[72]	Low	Low	Low	Low	Low	Low
[50]	Low	Low	Some concern	Low	Some concern	Some Concern
[73]	High	Low	Some concern	Some concern	Low	High
[32] trial 1	Some concern	Low	Some concern	Some concern	Low	Some Concern
[32]trial 2	Some concern	Low	Low	Some concern	Low	Some Concern
[32]	Some concern	Low	Some concern	Some concern	Low	Some Concern
[74]	Some concern	Low	Some concern	Some concern	Low	Some Concern
[29]	Some concern	Low	Some concern	Some concern	Low	Some Concern
[75]	High	Low	Low	Some concern	Low	High
[76]	Some concern	Low	Low	Some concern	Some concern	Some Concern
[31]	Some concern	Low	Some concern	Some concern	Low	Some Concern
[77]	Some concern	Low	Some concern	Some concern	Low	Some Concern
[33] trial 1	Some concern	Low	Some concern	Some concern	Low	Some Concern
[33] trial 2	Some concern	Low	Some concern	Some concern	Low	Some Concern
[35] trial 1	Some concern	Low	Low	Some concern	Some concern	Some Concern
[35] trial 2	Some concern	Low	Low	Some concern	Some concern	Some Concern
[35] trial 3	Some concern	Low	Low	Some concern	Some concern	Some Concern
[35] trial 4	Some concern	Low	Low	Some concern	Some concern	Some Concern
[79]	Some concern	Low	Low	Some concern	Low	Some Concern
[80]	Some concern	Low	Low	Some concern	Low	Some Concern
[36] trial 1	Some concern	Low	Low	Some concern	Some concern	Some Concern
[36] trial 2	Some concern	Low	Low	Some concern	Some concern	Some Concern
[81] trial 1	Some concern	Low	Some concern	Some concern	Low	Some Concern

[81] Trial 2	Some concern	Low	Low	Some concern	Low	Some Concern
[37]	Some concern	Low	Low	Some concern	Low	Some Concern
[38]	Some concern	Low	Some concern	Some concern	Low	Some Concern
[82] trial 1	Some concern	Low	Low	Some concern	Low	Some Concern
[82] trial 2	Some concern	Low	Low	Some concern	Low	Some Concern
[83]	Some concern	Low	Some concern	Some concern	Low	Some Concern
[84]	Some concern	Some concern	Low	Some concern	Some concern	Some Concern
[40]	Some concern	Low	Low	Some concern	Low	Some Concern
[39]	Some concern	Low	Low	Some concern	Low	Some Concern
[41]	Some concern	Low	Low	Some concern	Low	Some Concern
[42]	Some concern	Low	Low	Some concern	Low	Some Concern
[43]	Some concern	Low	Low	Some concern	Low	Some Concern
[45]	Some concern	Low	Low	Some concern	Low	Some Concern
[85]	Some concern	Low	Low	Some concern	Low	Some Concern
[47]	Some concern	Low	Some concern	Some concern	Some concern	Some Concern
[48] Trial 1	Some concern	Low	Low	Some concern	Some concern	Some Concern
[48] Trial 2	Some concern	Low	Low	Some concern	Some concern	Some Concern
[48] Trial 3	Some concern	Low	Low	Some concern	Some concern	Some Concern
[49]	Some concern	Low	Some concern	Some concern	Low	Some Concern
[86]	Some concern	High	Low	Some concern	Low	High
[51]	Some concern	Some concern	Some concern	Some concern	Low	Some Concern
[87]	Some concern	Some concern	Some concern	Some concern	Some concern	Some Concern
[52]	Some concern	Some concern	Some concern	Some concern	Some concern	Some Concern
[53]	Some concern	Low	Low	Some concern	Low	Some Concern
[30]	Some concern	Low	Low	Some concern	Low	Some Concern
[46]	Some concern	Low	Low	Some concern	Low	Some Concern

[88]	Some concern	Low	Low	Some concern	Low	Some Concern
[89]	Some concern	Some concern	Some concern	Some concern	Some concern	Some Concern
[44]	Some concern	Low	Low	Low	Low	Some Concern
[34]	High	Some concern	Some concern	Some concern	Some concern	High
[90]	Some concern	Some concern	Some concern	Some concern	Some concern	Some Concern

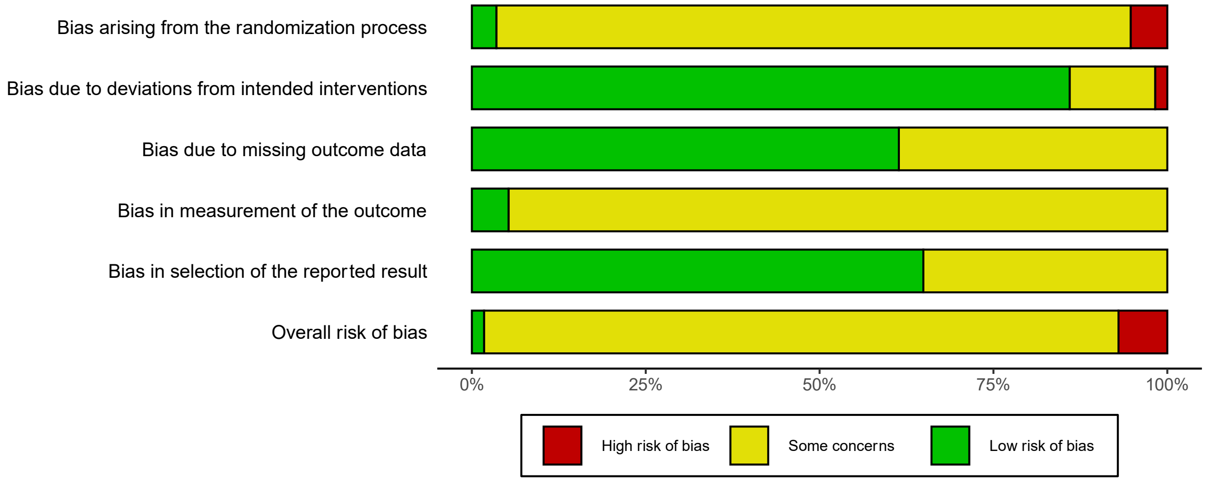

Figure S1. Risk of Bias assessment summary for 46 eligible studies

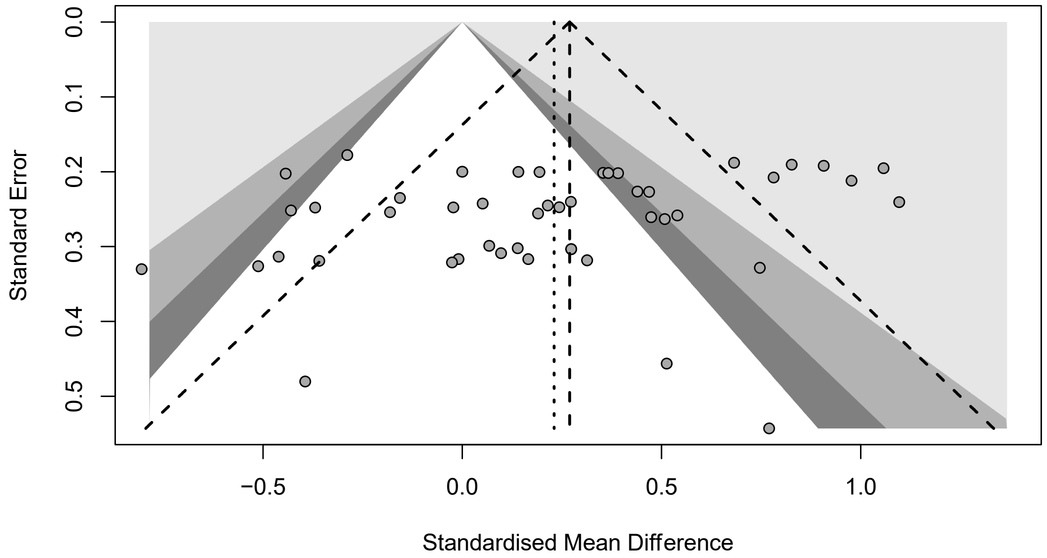

Figure S2. Contour enhanced funnel plot on studies examining stillbirth outcomes

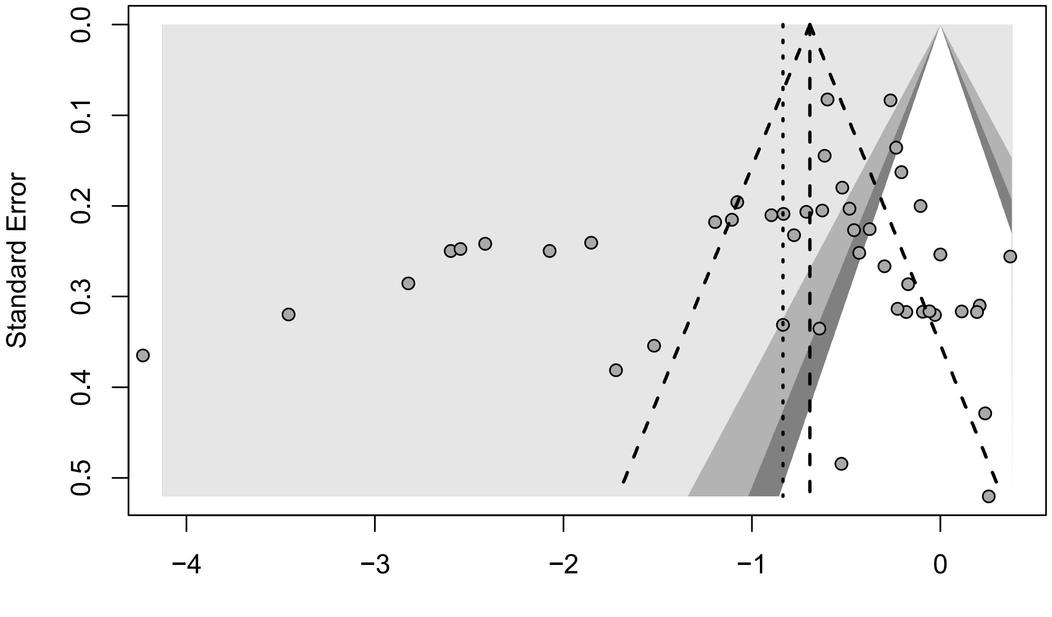

Figure S3. Contour enhanced funnel plot on studies examining farrowing duration

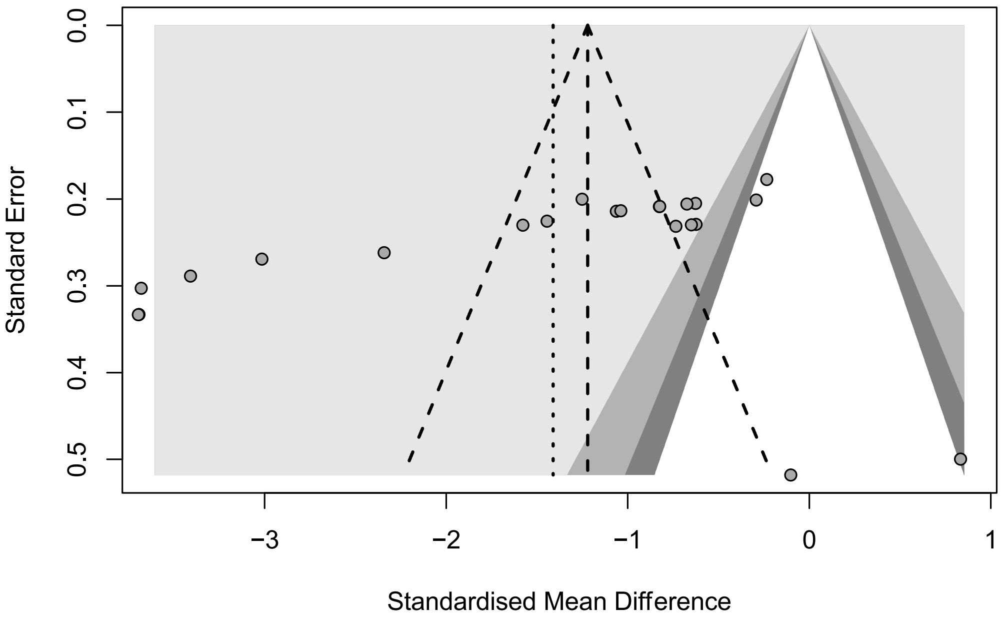

Figure S4. Contour enhanced funnel plot on studies examining time interval between piglets

Table S9. GRADE assessment summarized by domain for each outcome

**Outcome (No. of studies)**	**Risk of bias in individual studies**	**Inconsistency**	**Indirectness**	**Imprecision**	**Publication bias**	**Effect summary**	**Quality**
Stillbirths (19)	No serious limitations	Plausible explanation	Indirect comparison to dose (-1)	No serious imprecision	Undetected	SMD = 0.23 95CI= 0.10; 0.36 p<0.01	Moderate
Farrowing duration (22)	No serious limitations	Plausible explanation	Indirect comparison to dose (-1)	No serious imprecision	Undetected	SMD = -0.84 95CI= -1.07; -0.60 p<0.01	Moderate
Interval between piglets (11)	No serious limitations	Plausible explanation	Indirect comparison to dose (-1)	No serious imprecision	Undetected	SMD = -1.41 95CI= -1.86; -0.97 p<0.01	Moderate

Protocol.

Defining the efficacy of oxytocin use on farrowing sows and stillbirth rate: a protocol for a systematic review and meta- analysis

Registration

The systematic review protocol will be registered with the University of Guelph institutional repository (Atrium). The following protocol follows the Preferred Reporting Items for Systematic review and Meta-Analysis Protocols (PRISMA-P) 2015 checklist and the PRISMA-P Explanation and Elaboration (Moher *et al., 2015).*

Sarah V. Hill[1], Maria Amezcua[1], Eduardo Ribeiro[2], Terri O'Sullivan[1], Robert M. Friendship[1]

*Corresponding author: Sarah V. Hill shill09@uoguelph.ca

[1]Department of Population Medicine, Ontario Veterinary College, University of Guelph, Guelph, ON, Canada, N1G 2W1
[2]Department of Animal Bioscience, Ontario Agricultural College, University of Guelph, Guelph, ON, Canada, N1G 2W1

Author Contact:
Sarah V. Hill – shill09@uoguelph.ca; Maria Amezcua – mamezcua@uoguelph.ca; Eduardo Ribeiro – eribeiro@uoguelph.ca; Terri O'Sullivan – tosulliv@uoguelph.ca; Robert M. Friendship – rfriends@ovc.uoguelph.ca

Author Contributions:
S.V.H is the guarantor. S.V.H developed and wrote the protocol with input and revisions from all other authors. R.M.F and T.O. provided expertise in on swine health management. T.O provided expertise on statistical methodology. E.R. provided expertise on animal reproduction. S.V.H and M.A were the readers for the screening and data extraction process. R.M.F was the third reader for when there are discrepancies between the two readers. All authors read, provided feedback and approved the protocol prior to registration.

Amendments
If any amendments are made following the registration of this protocol, they will be documented and included in the final systematic review as protocol revisions.

Support

Sources

S.V. Hill's PhD program has been supported through the Ontario Veterinary College (OVC) PhD Scholarship and the Gallant Custom Laboratories Anniversary Scholarship.

Sponsors

There are no sponsors for this review.

Role of sponsor and/or funder

The sources for S.V. Hill's PhD program, are not involved in any stage of this review.

Rationale

Over the past 20 years, a lot has changed in pig farming including rapid genetic advances in selecting hyperprolific sows (Ward et al., 2020; Lukovic & Škorput, 2015; Rutherford et al., 2013). However, larger litter sizes can have ramifications which can affect the sow and the piglets during the farrowing process. Larger litter sizes increase duration of farrowing, increasing the likelihood of stillbirths, dystocia and intrapartum hypoxia (Ward et al., 2020; Oliviero et al., 2019).

Oxytocin is an important hormone for sows during farrowing. The hormone is produced in the hypothalamus and released from the pituitary glands during parturition (Linneen et al., 2005). Oxytocin release has two important functions: stimulation of uterine contractions and stimulate milk let down (Linneen et al., 2005). Administration of exogenous oxytocin is a common treatment for sows experiencing prolonged farrowing and agalactia (Linneen et al., 2005). According to oxytocin labels, administration of oxytocin can also be used to induce labour, accelerate the parturition process and expel postpartum uterine debris (Oxtyo-Sure, Vetoquinol; Oxy-20 NW, Rafter 8). In addition to numerous uses, its dosage range is quite wide. For the purpose of farrowing, the dose of exogenous oxytocin producers can use range between 1.5mL – 2.5mL (30 – 50 oxytocin units) (Oxtyo-Sure, Vetoquinol: Oxy-20 NW, Rafter 8).

Some research has shown that the misuse of oxytocin can result in complications including piglet asphyxiations and stillbirth (Linneen et al., 2005). In a survey done in the UK, researchers reported that 74% of respondents used oxytocin at least "sometimes" during farrowing and 54% of respondents used oxytocin at least "sometimes" after farrowing (Ison, Jarvis, & Rutherford, 2016). With the methodology of a systematic review and meta-analysis, the goal is to investigate the current literature on oxytocin use to define proper guidelines for oxytocin use on farrowing sows.

Objectives: The objective of this protocol is to define the methodology for a systematic review and meta-analysis. The aim of this systematic review and meta-analysis is to create a more stringent guideline for oxytocin use during farrowing which can be used by producers. The specific questions that will be addressed in the systematic review are:

1. When examining sows farrowing, were there negative side-effects to sows that received exogenous oxytocin compared to those that didn't receive exogenous oxytocin.

2. When comparing sows that received exogenous oxytocin to sows that didn't receive exogenous oxytocin, what was the comparative effectiveness to reducing stillbirths and improving piglet viability.

3. For questions 1 and 2, identify dosages, sow parity and reason for oxytocin administration.

Methods

Eligibility criteria:

i. **Population:** Sows either immediately before, during or immediately after farrowing stage
ii. **Intervention/Exposure:** Oxytocin given to sows either immediately before, during or immediately after farrowing stage
iii. **Comparator:** Sows either immediately before, during or immediately after farrowing stage not given any oxytocin or given the analogue- Carbetocin. As well as different sows given dosages and or different timing of administrations (early or late during farrowing process).
iv. **Outcomes:** Stillbirths, sow mortality, and piglet viability (such as mortality rate within the first few days, meconium staining, rupture umbilical cords).

Publication date: Articles must have been published within the last 50 years (1970 - present.
Report characteristics: The articles must be published in English. The articles can either be published or non-published but must be available in full text.
Study design: Only articles with clinical trial study designs will be included.

Information Sources: The search will include a range of relevant databases to identify peer-reviewed literature. Table S10 represents the databases used.

Table S10: Databases used to search for relevant literature

Database	Interface	Query Box	Wildcards
PubMed	National Center for Biotechnology Information (NCBI)	All fields	* - any group of characters<br>Note: wildcard can only be used if word is 4 or more characters
CAB Direct	CAB Interface (CABI)	All fields	? - 0 to 1 character<br>* - any group of characters
Web of Science Core Collection	Web of Knowledge	Topic (title, abstract, author keywords and Keyword Plus ®)	$ - 0 to 1 character<br>* - any group of characters
ProQuest dissertations and theses global (1988 - present)	ProQuest	Anywhere except full text	? - 0 to 1 character<br>* - any group of characters

In addition, a manual search in the table of contents for the following relevant conferences and reports in the American Association of Swine Veterinarian database including:

- AASV Annual Meeting (1999-2020)
- AASV Pre-Conference Seminars (2007-2019)
- Allen D. Leman Swine Conference (1998-2019)
- George A. Young Swine Health and Management Conference (1999-2012)
- International Pig Veterinary Society Congress (2000, 2002, 2004, 2006, 2008, 2010, 2012, 2014, 2016, 2018)
- International Symposium on Swine Disease Eradication (2001-2002, 2004)
- ISU Swine Disease Conference for Swine Practitioners (1999-2019)
- Journal of Swine Health and Production (1993-2020)

Search strategy: The search strategy used for this review was developed using key concept terms and words. Population, Intervention and Outcome terms will be connected using Boolean operators 'AND' and 'OR'. Table S13 represents an example of the search string that will be used for this review. The method used to develop the

search string has been illustrated below (Table S11, S12 and S13). To acquire more data from abstracts with no full-text and conference proceedings, S.V.H will contact the corresponding author. The two readers will do a final check for all relevant articles by reviewing the references of all articles which passed the two screening stages.

Table S11: Results of initial search string to identify articles investigating oxytocin use at farrowing and stillbirth rates, published between 1970 - present using Web of Science core collection database as of July 23/2020.

#	Search Terms	Results
1	(swine OR sow$ OR porcine OR pig$ OR gilt$)	440, 312
2	(oxytocin OR carbetocin)	27, 205
3	(stillbirth OR dystocia OR farrow* OR "fetal expulsion" OR intrapartum OR parturition OR stillborn OR birth OR meconium OR mortality OR hypoxia)	1,467,554
4	1 AND 2	973
5	3 AND 4	282

To ensure all keywords have been selected appropriately, a quick search on controlled vocabulary terms were done on MeSH 2020 browser and CAB thesaurus (Table S12). New terms were added to the search string (Table S13).

Table S12: Controlled vocabulary terms for PICO keywords

Key Term	MeSH 2020 browser:	CAB thesaurus
**Population :**<br>Pigs	Sus scrofa	Sus domesticus<br>Sus scrofa domesticus
**Intervention:**<br>Oxytocin*<br>Carbetocin*	N/A<br>N/A	N/A<br>N/A
**Outcome:**<br>Stillbirth	Fetal death	Fetal death

Dystocia<br><br>Hypoxia<br><br><br><br><br>Mortality	Uterine inertia<br><br>Anoxia<br>Anoxemia<br>Hypoxemia<br><br>Fetal Mortality	Parturition complications<br><br>Anoxia<br><br><br><br><br>Neonatal mortality<br>Perinatal mortality
* Oxytocin and carbetocin names are specified for animal use - no synonyms necessary<br>**Web of science has no controlled vocabulary or assignment of subject terms<br>*** Proquest uses MeSH 2020 thesaurus		

Table S13: Results of search string with addition of controlled vocabulary, published between 1970 - present using Web of Science core collection database as of July 23/2020

#	Search Terms	Results
1	(swine OR sow$ OR porcine OR pig$ OR gilt$ OR "sus scrofa" OR "sus domesticus" OR "sus scrofa domesticus")	442, 036
2	(oxytocin OR carbetocin)	27, 205
3	(stillbirth OR dystocia OR farrow* OR "fetal expulsion" OR intrapartum OR parturition OR stillborn OR birth OR meconium OR mortality OR hypoxia OR "fetal death" OR "uterine inertia" OR "parturition complications" OR anoxia OR anoxemia OR hypoxemia OR "fetal mortality" OR "neonatal mortality" OR "perinatal mortality")	1,491,102
4	1 AND 2	973
5	**3 AND 4**	**284**

Data Management: The resulting articles from the search string will be downloaded into a bibliographic software program (EndNoteWeb, Clarivate Analytics) or (Mendeley Ltd, Elsevier). Then all citations will be loaded onto DistillerSR where the following screening and data collection will be done. Prior to screening, all duplicates will be removed in Distiller SR.

Selection Process: The protocol for this review will have two stages of screening. The first stage will screen papers which were the result of the search string by assessing the relevance of the title and abstract.

1) "Is the title and abstract available in English?"
2) "Does the title and/or abstract mention exogenous oxytocin or Carbetocin use on sows?"
3) "Does the title and/or abstract mention infectious causes of stillbirths?"

The response to these questions will include YES, NO and UNCLEAR. A reference will be excluded if the reader identifies a NO to questions 1-3. A reference will be excluded if the reader identifies a YES to question 3. If there are any discrepancies between the two readers, a third reader will evaluate the title/abstract. If a reader responds with UNCLEAR for any of the questions, a third reader will evaluate the title/abstract.

The second stage of screening will determine the relevance of each article by assessing the full-text .

1) "Is the full text available in English?"
2) "Is the text more than 500 words
3) "Does the article discuss the use of exogenous oxytocin for farrowing purposes?"
4) "Does the full-text focus on infectious causes of stillbirths?"
5) "Is there a comparator group (either Carbetocin or no oxytocin)?"
6) "Is the study design a trial"

The response to these questions will include YES, NO and UNCLEAR. A reference will be excluded if reader identifies a NO to questions 1-3, 5 and 6. A reference will be excluded if reader identifies a YES to question 4. If there are any discrepancies between the two readers, a third reader will evaluate the full-text.

Data collection process: The eligible studies will be further processed for data collection. The two readers will use a standardized form through DistillerSR to ensure consistency in data extraction.

Data items: The following information will be collected:
Study information: year of Publication, year (or range) study was conducted, month(s) study was conducted, and country.
Population information: research or commercial herd, breed of sows, number of sows and parity of sows.
Intervention and comparator information: Name of exogenous oxytocin used, reason for oxytocin use, perform manual assistance, supervision during farrowing, dosage, number of administrations per sow and combine oxytocin with pain medication.

Outcomes and prioritisation:
Outcome data to be extracted:

- Stillbirth rate
  a) Total piglets born, piglets born alive and dead
  b) Farrowing order
- Mean interval time between piglets in minutes
- Mean duration time of farrowing in minutes
- Meconium staining of piglet (degree of staining)
  a) Mild, medium, severe
- Umbilical cord appearance?
  a) Adhered or ruptured
- Sow behaviour
  a) Nesting behaviour, eating and drinking, position in crate
- Sow mortality

Risk of bias individual studies
The Cochrane tool for Risk of Bias in Randomized studies of interventions (RoB 2.0) will be used for assessing the risk of bias for each study (Sterne et al., 2019). Assessing the risk of bias for individual studies will be done at the outcome level. The assessment will be performed independently by two readers. Using the RoB assessment tool, there are a fixed set of domains being used to assess the overall risk of bias. The following set of domains of bias include: confounding(bias due to either unmeasured or uncontrolled variable which predicts whether individual receives intervention of interest), selection (bis due to selection of participants into study and/or bias due to missing data), information (measurement error in outcome status or measurement error in exposure status) and reporting bias (selection of outcome measure, type of analysis and/or selective reporting (Sterne et al., 2019). The RoB assessment tool which consists of a series of signalling questions. Based on the responses to the questions, an overall risk of bias will be determined: 'Low', Moderate', 'Some Concerns'(Sterne et al., 2019).

Data synthesis

Prior to conducting a meta-analysis, the readers will confirm that the studies are homogenous in terms of study design and comparators. If meta-analysis is deemed inappropriate due to study heterogeneity, a qualitative narrative summary will be done instead. This would entail descriptive text and tables summarizing study characteristics and findings. Each meta-analysis will be visualized using a forest plot. For the meta-analysis, data synthesis will be done for each outcome separately. The goal is to assess the direction of effect for each outcome, the sized of effect and whether the effect is consistent across studies (Deeks et al., 2011). Finally, readers will assess the strength of evidence for effect by assessing study design and risk of bias (Deeks et al., 2011). Dichotomous data will be analyzed using an risk ratio (with 95% confidence interval) of an event occurring. Continuous data will be analyzed using the mean difference approach. Possible sources of heterogeneity include parity of the sow, oxytocin combined with pain medication, supervision and manual assistance. Subgroup analysis will be performed on these possible sources of heterogeneity.

Meta-bias(es)

To reduce reporting bias, a funnel plot will be assessed for each outcome synthesized with 10 or more studies.

Confidence in cumulative evidence

The strength of evidence for each outcome will be assessed using the Grading of Recommendations Assessment, Development and Evaluation (GRADE) methodology (Guyatt et al., 2008). This systems assesses the risk of bias, publication bias, precision, directness and consistency for each outcome (Guyatt et al., 2008).

Reference

1. Ward, S.A.; Kirkwood, R.N.; Plush, K.J. Are Larger Litters a Concern for Piglet Survival or an Effectively Manageable Trait? Animals 2020, 10, 309. https://doi.org/10.3390/ani10020309.
2. Oliviero, C.; Junnikkala, S.; Peltoniemi, O. The Challenge of Large Litters on the Immune System of the Sow and the Piglets. Reprod. Domest. Anim. 2019, 54, 12–21. https://doi.org/10.1111/rda.13463.
3. Linneen, S.K.; Benz, J.M.; DeRouchey, J.M.; Goodband, R.D.; Tokach, M.D.; Dritz, S.S. A Review of Oxytocin Use for Sows and Gilts. Kans. Agric. Exp. Stn. Res. Rep. 2005, 10, 1–3. https://doi.org/10.4148/2378-5977.6934.
4. Guyatt, G.H.; Oxman, A.D.; Vist, G.E.; Kunz, R.; Falck-Ytter, Y.; Alonso-Coello, P.; Schünemann, H.J. GRADE: An Emerging Consensus on Rating Quality of Evidence and Strength of Recommendations. BMJ 2008, 336, 924–926. https://doi.org/10.1136/bmj.39489.470347.AD.
5. Sterne, J.A.C.; Savović, J.; Page, M.J.; Elbers, R.G.; Blencowe, N.S.; Boutron, I.; Cates, C.J.; Cheng, H.-Y.; Corbett, M.S.; Eldridge, S.M.; et al. RoB 2: A Revised Tool for Assessing Risk of Bias in Randomised Trials. BMJ 2019, 366, l4898. https://doi.org/10.1136/bmj.l4898.
6. Ison, S.; Jarvis, S.; Rutherford, K. A Survey of Sow Management at Farrowing in the UK. Anim. Welf. 2016, 25, 309–317. https://doi.org/10.7120/09627286.25.3.309.
7. PRISMA-P Group; Moher, D.; Shamseer, L.; Clarke, M.; Ghersi, D.; Liberati, A.; Petticrew, M.; Shekelle, P.; Stewart, L.A. Pre-ferred Reporting Items for Systematic Review and Meta-Analysis Protocols (PRISMA-P) 2015 Statement. Syst. Rev. 2015, 4, 1. https://doi.org/10.1186/2046-4053-4-1.
8. Deeks, J.J.; Higgins, J.P.T.; Altman, D.G.; Green, S. (Eds.) Chapter 9: Analysing data and undertaking meta-analyses. In Cochrane Handbook for Systematic Reviews of Interventions Version 5.1.0; Updated March 2011; The Cochrane Collaboration, London, UK: 2011. Available online: www.handbook.cochrane.org (accessed on 25 May 2022).
9. Lukovic, Z.; Škorput, D. Factors influencing litter size in pigs. CAB Rev. 2015, 10, 1–9.
10. Rutherford, K.M.D.; Baxter, E.M.; D'Eath, R.B.; Turner, S.P.; Arnott, G.; Roehe, R.; Ask, B.; Sandøe, P.; Moustsen, V.A.; Thorup, F.; et al. The welfare implications of large litter size in the domestic pig I: Biological factors. Anim. Welf. 2013, 22, 199–218.
11. Shamseer, L.; Moher, D.; Clarke, M.; Ghersi, D.; Liberati, A.; Petticrew, M.; Shekelle, P.; Stewart, L.; PRISMA-P Group. Preferred reporting items for systematic review and meta-analysis protocols (PRISMA-P) 2015: Elaboration and explanation. BMJ 2015, 349, g7647.
12. Boulot, S.; Dubroca, S.; Quiniou, N.; Charpiat, O.; Ruelland, P.Y. Effect of carbetocin at the onset of parturitionon farrowing duration and piglet traits. In Proceedings of

the 19th International Pig Veterinary Society Congress, Copenhagen, Denmark, 16–19 July 2006; Volume 2, p. 514.
13. Carlo, C.; Alessandro, P.; Fausto, G.; Paolo, B.; Paolo, B.; Lucio, N.P. Assessment of sow births synchronised with oxytotocin and long-acting oxytocin (carbetocin). In Proceedings of the 21st International Pig Veterinary Society Congress, Vancouver, Can-ada, 18–21 July 2010; p. 1106.
14. Cameron, R.D.A.; Kieran, P.J.; Martin, I. The efficacy in inducing batch farrowing and the impact on sow behaviour of the prostaglandins cloprostenol and dinoprost. In Proceedings of the 16th International Pig Veterinary Society Congress, Mel-bourne, Australia, 17–21 September 2000; p. 386.
15. Mota, D.; Marinez-Brunes, J.; Trujullo, M.E.; Alonso-Spilsbury, M.; Ramirez-Necoechea, R.; Lopez-Mayagoitia, A. Use of Ox-ytocin during Farrowing: Effects on the Umbilical Cord and Neonatal Deaths in Pigs; p. 685.
16. Alexopoulos, C. Association of Fusarium mycotoxicosis with failure in applying an induction of parturition program with PGF2alpha and oxytocin in sows. Theriogenology 2001, 55, 1745–1757.
17. Boonraungrod, N.; Sutthiya, N.; Kumwan, P.; Tossakui, P.; Nuntapaitoon, M.; Muns, R.; Tummaruk, P. Gestation length, farrowing time and neonatal piglet characteristics after induction of parturition by using PGF2α in combination with carbetocin in sows. Thai J. Vet. Med. 2016, 46, 199–200.
18. Boonraungrod, N.; Sutthiya, N.; Kumwan, P.; Tossakui, P.; Nuntapaitoon, M.; Muns, R.; Tummaruk, P. Control of parturition in swine using PGF2α in combination with carbetocin. Livest. Sci. 2018, 214, 1–8.
19. Chantaraprateep, P.; Prateep, P.; Lohachit, C.; Poomsuwan, P.; Kunavongkrit, A. Investigation into the use of prostaglandin F2α (PGF2α) and oxytocin for the induction of farrowing. Aust. Vet. J. 1986, 63, 254–256.
20. Decaluwé, R.; Janssens, G.P.J.; Englebienne, M.; Maes, D. Effectiveness of different farrowing induction protocols in sows using alphaprostol on day 114 of gestation. Vet. Rec. 2014, 174, 381.
21. Holtz, W.; Schmidt-Baulain, R.; Meyer, H.; Welp, C. Control of prostaglandin-induced parturition in sows by injection of the beta-adrenergic blocking agent carazolol or carazolol and oxytocin. J. Anim. Sci. 1990, 68, 3967–3971.
22. Kirkwood, R.N.; Aherne, F.X. Increasing the predictability of cloprostenol-induced farrowing in sows. Swine Health Prod. 1998, 6, 57–59.
23. Kirkwood, R.N.; Thacker, P.A. Effect of propranolol on the onset and duration of parturition in sows. Can. Vet. J. 1995, 36, 238–239.
24. Maffeo, G.; Vigo, D.; Ballabio, R.; Olivia, O.; Cairoli, F.; Jöchle, W. Uterine motility in sows during spontaneous parturition and induced parturitions with the PGF analog alfaprostol and oxytocin. Reprod. Domest. Anim. 1990, 25, 36–43.
25. Nacu, G.; Irimia, C.; Ciornei, Ș. Research regarding parturition synchronization in sows by using F2alpha prostaglandin and oxytocin. Lucr. Științifice-Univ. De Științe Agric. Și Med. Vet. Ser. Zooteh. 2014, 62, 9–12.

26. Too, H.L.; Vel, M. Induction of parturition in sows by vulvomucosal injection of cloprostenol. J. Vet. Malays. 1994, 6, 25–28.
27. Wehrend, A.; Stratmann, N.; Failing, K.; Bostedt, H. Influence of partus induction on the pH value in the blood of newborn piglets. J. Vet. Medicine. Ser. A 2005, 52, 472–473.
28. Hernandez, V.F.; Canseco, A.B.; Hernandez, J.R.O. Programmed Farrowing with Prostaglandin and Oxitocin in the Sow. J. Anim. Vet. Adv. 2009, 8, 1045–1048.
29. Wahner, M.; Huhn, U. Control of parturition in sows by using a combined treatment with Cloprostenol (R) plus Depotocin (R). Arch. Fur Tierz.-Arch. Anim. Breed. 2001, 44, 151–154.
30. Maffeo, G.; Geroldi, S.; Cerati, C.; Salvo, R.; Nisoli, L.G.C.; Colombani, C.; Vigo, D. The use of carbetocine in sows at the be-ginning of farrowing. In IPVS Reproduction, Breeding and Genetics; Bologna, Italy, 1996; p. 466.
